# The INNs and outs of antibody nonproprietary names

**DOI:** 10.1080/19420862.2015.1114320

**Published:** 2015-12-30

**Authors:** Tim D. Jones, Paul J. Carter, Andreas Plückthun, Max Vásquez, Robert G.E. Holgate, Isidro Hötzel, Andrew G. Popplewell, Paul W.H.I. Parren, Markus Enzelberger, Hendrik J. Rademaker, Michael R. Clark, David C. Lowe, Bassil I. Dahiyat, Victoria Smith, John M. Lambert, Herren Wu, Mary Reilly, John S. Haurum, Stefan Dübel, James S. Huston, Thomas Schirrmann, Richard A.J. Janssen, Martin Steegmaier, Jane A. Gross, Andrew R.M. Bradbury, Dennis R. Burton, Dimiter S. Dimitrov, Kerry A. Chester, Martin J. Glennie, Julian Davies, Adam Walker, Steve Martin, John McCafferty, Matthew P. Baker

**Affiliations:** 1 Antitope Ltd. (part of Abzena Plc.), Babraham Research Campus, Cambridge CB22 3AT, UK; 2 Genentech Inc., 1 DNA Way, South San Francisco, CA 94080, USA; 3 Department of Biochemistry, University of Zurich, Zurich CH-8057, Switzerland; 4 Adimab LLC., 7 Lucent Drive, Lebanon, NH 03766, USA; 5 UCB Pharma., 208 Bath Road, Slough SL1 3WE, UK; 6 Genmab, PO Box 85199, 3508 AD, Utrecht, The Netherlands; 7 Leiden University Medical Center, Department of Immunohematology and Blood Transfusion, Leiden University Medical Center, Albinusdreef 2, 2333 ZA Leiden, The Netherlands; 8 MorphoSys AG., Lena-Christ-Str. 48, 82152 Martinsried/Planegg, Germany; 9 Clark Antibodies Ltd., 11 Wellington Street, Cambridge CB1 1HW, UK; 10 MedImmune Ltd., Milstein Building, Granta Park, Cambridge CB21 6GH, UK; 11 Xencor., 111 West Lemon Avenue, Monrovia, CA 91016, USA; 12 Gilead Sciences Inc., Foster City, CA 94404, USA; 13 ImmunoGen Inc., 830 Winter Street, Waltham, MA 02451-1477, USA; 14 MedImmune., One MedImmune Way, Gaithersburg, MD 20878, USA; 15 Opsona Therapeutics Ltd., 2nd Floor, Ashford House, Tara Street, Dublin 2, Ireland; 16 F-Star Biotechnology Ltd., Babraham Research Campus, Cambridge CB22 3AT, UK; 17 Technische Universität Braunschweig., Institute of Biochemistry, Biotechnology and Bioinformatics Spielmannstr. 7, 38106 Braunschweig, Germany; 18 The Antibody Society & Huston BioConsulting LLC., 270 Pleasant Street #A206, Watertown, MA 02472, USA; 19 Yumab GmbH., Rebenring, 38106 Braunschweig, Germany; 20 Galapagos NV., Zernikedreef 16, 2333 CL Leiden, Netherlands; 21 Roche Pharmaceutical Research and Early Development,. Large Molecule Research, Roche Innovation Center Penzberg, 82377 Penzberg, Germany; 22 Emergent BioSolutions., 2401 4th Avenue, Suite 1050, Seattle, WA 98121, USA; 23 Biosciences Division., MS-M888, TA-43, HRL-1, Building 1, Los Alamos National Laboratory, Los Alamos, NM 87545, USA; 24 The Scripps Research Institute., 10550 North Torrey Pines Road, La Jolla, CA 92037, USA; 25 Protein Interactions Section., Cancer and Inflammation Program, Center for Cancer Research, National Cancer Institute, Frederick, MD 21702, USA; 26 UCL Cancer Institute., 72 Huntley Street, London WC1E 6BT, UK; 27 Antibody and Vaccine Group, Cancer Sciences Unit, University of Southampton Faculty of Medicine, Southampton General Hospital, Southampton, Hampshire SO16 6YD, UK; 28 Lilly Biotechnology Center San Diego, CA 92121, USA; 29 GSK., Addenbrooke's Centre for Clinical Investigation, Addenbrooke's Hospital, Hills Road, Cambridge, CB2 2GG, UK; 30 GSK, Medicines Research Centre, Gunnels Wood Road, Stevenage, Herts, SG1 2NY, UK; 31 Iontas Ltd., Babraham Research Campus, Cambridge CB22 3AT, UK

**Keywords:** antibody, chimeric, Complementarity Determining Region (CDR), definition, framework, humanized, International Nonproprietary Name (INN), International Immunogenetics Information System (IMGT), monoclonal, World Health Organization (WHO)

## Abstract

An important step in drug development is the assignment of an International Nonproprietary Name (INN) by the World Health Organization (WHO) that provides healthcare professionals with a unique and universally available designated name to identify each pharmaceutical substance. Monoclonal antibody INNs comprise a –mab suffix preceded by a substem indicating the antibody type, e.g., chimeric (-xi-), humanized (-zu-), or human (-u-). The WHO publishes INN definitions that specify how new monoclonal antibody therapeutics are categorized and adapts the definitions to new technologies. However, rapid progress in antibody technologies has blurred the boundaries between existing antibody categories and created a burgeoning array of new antibody formats. Thus, revising the INN system for antibodies is akin to aiming for a rapidly moving target. The WHO recently revised INN definitions for antibodies now to be based on amino acid sequence identity. These new definitions, however, are critically flawed as they are ambiguous and go against decades of scientific literature. A key concern is the imposition of an arbitrary threshold for identity against human germline antibody variable region sequences. This leads to inconsistent classification of somatically mutated human antibodies, humanized antibodies as well as antibodies derived from semi-synthetic/synthetic libraries and transgenic animals. Such sequence-based classification implies clear functional distinction between categories (e.g., immunogenicity). However, there is no scientific evidence to support this. Dialog between the WHO INN Expert Group and key stakeholders is needed to develop a new INN system for antibodies and to avoid confusion and miscommunication between researchers and clinicians prescribing antibodies.

## A Brief History of WHO INNs as Applied to Monoclonal Antibodies

International non-proprietary names (INNs) are generic and nonproprietary names assigned to drug substances that are unique to the active ingredient and are designed to enable accurate communication among healthcare professionals. In the case of small molecules, they help to abbreviate the very long systematic chemical names and give an indication of the function of a compound, whereas in the case of protein pharmaceuticals they give an indication of the nature of the protein. The INN system has been coordinated by the World Health Organization (WHO) since 1950 and the suffix ‘-mab’ was introduced as the stem name to describe monoclonal antibodies in 1990. Following on from this, a system of substems was derived to describe the antibody origin and disease/target indication^1^ with the allowance of a prefix chosen by the originator. The ‘origin’ substem was necessary to distinguish between different antibody formats, for example: mouse (-o-), chimeric (-xi-), humanized (-zu-) or human (-u-) and, therefore, a definition for each category was required in order to allow appropriate naming for new monoclonal antibody therapeutics. The 1997 definition^1^ of chimeric antibody was:

‘*one that contains contiguous foreign-derived amino acids comprising the entire variable region of both heavy and light chains linked to heavy and light constant regions of human origin*’;

and a humanized antibody was defined as:

‘*heavy (H) and light (L) chain variable (V) regions, consisting of the amino acids comprising the complementarity-determining region (CDR) segments (and possibly frameword* [sic] *residues) from foreign antibodies inserted appropriately among variable regions framework segments of human-derived amino acid residues, linked to H and L constant regions of human origin*’.

Subsequently, in 2009, the definition of a humanized antibody was updated^2^ to a more concise description without substantially changing the meaning or composition of a humanized antibody, compared to the 1997 definition (the chimeric definition remained the same). The definition was as follows:

‘*A humanized antibody has segments of foreign-derived amino acids interspersed among variable domain segments of human-derived amino acid residues and the humanized variable heavy and variable light domains are linked to heavy and light constant regions of human origin*’.

Both the 1997 and 2009 definitions of a humanized antibody allowed for the inclusion of foreign complementarity-determining regions (CDRs) and additional framework residues (although not explicitly stated in the 2009 definition as in the 1997 definition). Definitions up to this point were largely consistent with the scientific literature from the last 25 to 30 years, and contributed to the creation of an implicit hierarchy of better to worse, as one goes from human to humanized to chimeric to mouse.

The chimeric and humanized antibody definitions were again updated in 2011^3^ such that:

‘*A chimeric antibody is one of which both chain types are chimeric as a result of antibody engineering. A chimeric chain is a chain that contains a foreign variable domain (V-D-J-REGION) (originating from one species other than human, or synthetic) linked to a constant region (C-REGION) of human origin*’;

‘*A humanized antibody is one of which both chain types are humanized as a result of antibody engineering. A humanized chain is a chain in which the complementarity determining regions (CDR) of the variable domains are foreign (originating from one species other than human, or synthetic) whereas the remaining chain is of human origin*. *By extension an antibody is described as humanized if more recent protocoles* [sic] *were used for the humanization.’*

These definitions introduced the concept of synthetic sequences, i.e., sequences that are artificially created. The chimeric definition remained clear in that non-human variable region sequences were fused to human constant regions. In contrast, the humanized definition had changed significantly in that it only considered a sequence to be humanized if the CDRs alone had been transferred to human frameworks regions. This position is qualified by the final sentence that creates ambiguity as to whether or not an antibody that contains additional foreign framework residues would qualify as “humanized” since it is not clear what “more recent protocols” means.

In the most recent (2014) update to the WHO INN definitions,^4^ the grey area between chimeric and humanized antibodies is handled in a different way and the concept of comparing sequences ‘as a whole’ is introduced as follows:

‘*A chimeric antibody is one for which both chain types are chimeric as a result of antibody engineering. A chimeric chain is a chain that contains a foreign variable domain (originating from one species other than human, or synthetic or engineered from any species including human) linked to a constant region of human origin. The variable domain of a chimeric chain has a V region amino acid sequence which, analyzed as a whole, is closer to non-human species than to human*’;

‘*A humanized antibody is one for which both chain types are humanized as a result of antibody engineering. A humanized chain* is *typically a chain in which the complementarity determining regions (CDR) of the variable domains are foreign (originating from one species other than human, or synthetic) whereas the remainder of the chain is of human origin. Humanization assessment is based on the resulting amino acid sequence, and not on the methodology per se, which allows protocols other than grafting to be used. The variable domain of a humanized chain has a V region amino acid sequence which, analyzed as a whole, is closer to human than to other species'*

These latest definitions for humanized antibodies are not directly influenced by how the antibody sequences are obtained and therefore accommodate the many novel ways of humanizing a non-human antibody sequence. They also accommodate the fact that the distinction between CDRs and frameworks is artificial and man-made, primarily through interpreting the 3D structure and regions of diversity between antibodies. In all of the earlier definitions, the designation of a humanized antibody is confused by how CDRs are defined as there are 3 main systems for doing so, based upon either sequence variability (the Kabat system),^5^ the inclusion of structural data (the Chothia system)^6^ or strict adherence to the structural location of loops between β sheets (the IMGT® system).^7^ This issue has been resolved by the stipulation that sequences be analyzed “as a whole” and categorization is based upon the highest level of identity such that a humanized antibody must be more similar to human sequences than other species. While the definition of “humanized” still contains the conceptual roots of CDR grafting, the introduction of the word “typically” indicates that transfer of residues outside CDRs to the humanized antibody is not anticipated, but is allowed, and the fact that only the final overall sequence is considered resolves the issue of defining CDRs and frameworks. Whether these definitions resolve the issue between chimeric and humanized antibodies depends on how the exact definition of “closer to human” over the whole antibody sequence is interpreted.

## General Inconsistencies within the Current Definitions

There are several major problems with the latest WHO INN definitions that have led to inconsistencies and unintended outcomes in naming therapeutic antibodies. As described above, the guidelines provide an outline of the requirements for obtaining a chimeric or humanized designation but they make it very difficult, if not impossible, for a researcher designing a humanized antibody to determine whether or not it will be considered humanized by the WHO when its INN is assigned. In particular this relates to the human sequence database used for analysis and how that analysis is done. At an open session of the WHO INN Expert Group in April 2015 it was clarified that the comparison to human sequences should be done through the International Immunogenetics Information System® (IMGT®) DomainGapAlign tool (www.imgt.org). DomainGapAlign is a tool similar to BLAST^8^ which interrogates the IMGT® database of antibody germline variable region genes where the alignment score is made only against germline sequence variable region exons, thus omitting part of CDR3 and the J region from the analysis. As well as being “closer to human than to other species” it was also explained that the top “hit” must be human and, notwithstanding the results of this analysis, the identity to human sequences must be ≥ 85%, otherwise the antibody will be designated as chimeric. Unfortunately, these critical parameters are not included in the above definitions and are only, as far as we are aware, available via the American Medical Association^9^ requiring the registration of a free account.

With respect to the identity threshold, the rationale for using a score of similarity to the human germline sequence as a criterion for defining therapeutic antibodies has not been clearly stated. However, one interpretation is the notion that higher similarity may correlate with reduced immunogenicity.^10^ However, there is neither a scientific basis for a defined numerical threshold above which an antibody is guaranteed to be non-immunogenic, nor for the implicit assumption that the overall percentage identity is more important than the presence or absence of particular sequence features, which could act as T cell epitopes. By using an arbitrary dividing line in an ill-defined and unknown potential for immunogenicity, it appears as if the INN definition is attempting to provide quantitative clarity on immunogenicity where there is, in fact, none. Immunogenicity represents a complex and multi-factorial problem that is affected by many factors beyond sequence identity.^11,12^

Furthermore, the omission of the sequences encoded by the J-region genes should be considered a major flaw in the interpretation of the new rules, since the recombination of the V- and J-gene segments (and for the heavy chain the V-, D- and J-gene segments) creates the sequence that encodes the amino acids of the complete variable region domain; analysis of the variable domain sequence ‘as a whole’ should therefore include the J-region. The J-region makes a significant contribution to overall human germline identity for all engineered antibodies. Indeed it is particularly important to the humanization process where the J-region-encoded framework 4 of the parental antibody, in common with the other frameworks of the variable domain, is replaced by human sequences.

Another unintended consequence has arisen as a result of using the DomainGapAlign tool to align the humanized variable regions against the IMGT species database that also includes macaque variable region sequences among those from other species. The macaque variable region genes have a higher level of identity to the human genes than the other species in the IMGT database due to their relative evolutionary proximity, yet occasionally their CDR sequences can be closer to the parental species CDR (e.g., rat or mouse) than the human counterparts. This can give rise to the situation where the DomainGapAlign tool, which includes the CDRs in the identity calculation, gives the closest match to the humanized sequence as macaque, even though the frameworks share more sequence identity with the human and the CDRs share more sequence identity with the rodent. This point can be illustrated by analyzing the sequences of currently approved humanized antibodies where there are a number of instances of which the top “hit” is a macaque sequence, for example the light chain variable regions of vedolizumab, trastuzumab and tocilizumab. The notion that a CDR-grafting process from a rodent antibody onto human frameworks creates a chimeric macaque sequence is clearly misleading. This problem is further compounded by the fact that the composition of the database is not constant, and, with the addition of ever more complete genomes (and thus variable regions of other species), there is an increased likelihood that the top “hit” will be non-human.

## Impact of 2014 WHO INN Definition on Human Antibodies

The current WHO review on INN (ref. 4) provides a substem for human antibodies (-u-) but with no supporting definition. Thus, it is unclear if the -u- designation would be applied to human antibodies derived from a growing array of technologies. Interestingly, within the chimeric antibody definition (“engineered from any species, including human”), there is the possibility that human antibodies engineered for improved affinity or other properties could be considered chimeric. The American Medical Association^9^ provide some guidance (also adopted by the INN) on the criteria for defining antibodies derived from different methods e.g., isolation from various display libraries,^13,14^^,^^15,16^ use of animals transgenic for human antibody genes^17,18^^,^^19,20^^,^^21^ and direct isolation from humans^22,23^^,^^24,25^^,^^26^ where identity to human sequences must be ≥85%. However, this threshold has recently changed from a previous version that stipulated ≥90%. The reason for this change is unclear; however, it may be due to the fact that a number of approved human antibodies did not meet the original criteria but now meet the ≥85% identity threshold (**Table 1**).

**Table 1.** Analysis of the sequences of 14 approved ‘fully human’ antibodies using DomainGapAlign. Values in bold do not meet the ≥ 90% threshold and also overlap with the range of values found with humanized antibodies (see Table 2)

	Variable Heavy	Variable Light	
	% Human Identity	% Human Identity	Current WHO INN Designation
Panitumumab^a^	**89.9**	95.8	Human
Adalimumab^b^	93.9	95.8	Human
Canakinumab^a^	93.9	98.9	Human
Raxibacumab^b^	99.0	100.0	Human
Ipilimumab^a^	94.9	97.9	Human
Belimumab^b^	**86.7**	97.9	Human
Denosumab^a^	98.0	95.8	Human
Nivolumab^a^	91.8	98.9	Human
Secukinumab^a^	92.9	100.0	Human
Ramucirumab^b^	99.0	**85.3**	Human
Ustekinumab^a^	**87.8**	98.9	Human
Ofatumumab^a^	97.0	100.0	Human
Golimumab^a^	94.9	98.9	Human
Alirocumab^a^	**89.8**	94.1	Human
Evolocumab^a^	93.9	95.9	Human

a Antibody isolated from transgenic mouse;

b Antibody isolated from a phage display library.

As highlighted by**Table 1**, and implicitly acknowledged by the reduction of the identity threshold for human antibodies, the distinction between human antibodies and humanized antibodies is somewhat blurred albeit with a clear trend for human antibodies to have higher human germline gene identities. The problem with reducing the threshold to ≥ 85% is that this eliminates any sequence distinction between humanized and human antibodies. Consequently, the -zu- and -u- substems have become ambiguous and effectively redundant. In this case the antibody can only be categorized by the method used to obtain it, rather than the resultant sequence, which goes against the purpose of the new INN definitions.

In addition to the arguments related to immunogenicity in the previous section, there is no scientific rationale for the arbitrary ≥ 85% identity to human sequence threshold for “human” antibodies since even antibodies derived from human patients may fail this test due to high levels of somatic hypermutation; for example the PGT121 series of anti-HIV antibodies in which variable domains were derived in their entirety from human patients^27^ provide identity scores as low as 71% for the variable heavy chains using the recommended DomainGapAlign system (the tool is even unable to appropriately align the variable light chains) and the patient-derived antibody 3BNC60^28,29^ has identities as low as 58.8% and 69.9% in VH and VL chains, respectively. Clearly, each of these antibodies is human by origin, and thus they cannot be “chimeric.” However, as discussed above, the stated and reasonable purpose of the new INN rules is that the final outcome determines antibody designation rather than the engineering history. Therefore, a new and unintended inconsistency is created. Indeed the conflict of antibody sequence origin versus designation may also arise with antibodies derived from human libraries and transgenic mice with human-like IgG repertoires, which all evolve under similar selection pressures: form follows function.

## Impact of 2014 WHO INN Definition on Humanized Antibodies

For non-human antibodies that are humanized using contiguous human variable region germline framework templates with closest identity to the foreign antibody, meeting the ≥ 85% threshold is challenging. Indeed a vast body of experimental evidence (for examples see refs. 30,31,32,33,34,35 and for a review see ref. 36) demonstrates unequivocally that, for the majority of non-human antibodies, transfer of a small number of framework residues is required to reconstitute acceptable antigen binding and functional activity in their humanized counterparts. A further consequence of the “analyzed as a whole” concept is that it is unlikely that antibodies humanized using technologies in which non-contiguous human antibody fragments are used^37,38^^,^^39^ will be designated as humanized. This concept will also effectively penalize antibodies designed using fixed (including consensus) frameworks (e.g., trastuzumab^30^ and pertuzumab^31^) that are not selected based upon similarity to the original murine sequence. Under the new INN definitions such antibodies will be considered synthetic and will therefore be designated as chimeric; however, there is no scientific or clinical basis for making this distinction, as in fact trastuzumab has been shown to have a very low immunogenicity risk profile.^40^

**Table 2** shows an analysis of 16 currently licensed humanized antibodies based on the new WHO INN definitions (as clarified at the open session in April 2015) and reveals that, if the top “hit” rule is ignored, only 3 antibodies would be considered humanized, whereas 9 would be given a mixed designation (-xizu-, where one chain is humanized and the other is chimeric) and 4 would be chimeric. If the top “hit” rule is applied, then no antibodies would be designated humanized and a total of 9 would be considered chimeric.

**Table 2.** Sequence analysis of 16 approved humanized antibodies based upon the current WHO INN definitions using DomainGapAlign. Values in italics indicate criteria for humanized designation passed. Values in bold indicate criteria failed. Assumes decimals are rounded to the nearest whole number.

	Variable Heavy		Variable Light		
	% Mouse Identity	% Human Identity	% Mouse Identity	% Human Identity	New WHO INN Designation
Pembrolizumab^a^	72.4	**79.6**	79.8	*85.1*	Mixed
Vedolizumab^a^	81.6	*84.7*	85.0	*85.0*^e^	Humanized
Trastuzumab^b^	71.4	**81.6**	75.8	*86.3*^e^	Mixed
Obinutuzumab^c^	77.6	*84.7*	90.0	87.0^f^	Humanized
Pertuzumab^b^	72.4	**78.8**	77.9	**84.2**^e^	Chimeric
Tocilizumab^a^	77.3	*84.8*	83.2	*89.5*^e^	Humanized
Certolizumab^b^	70.6	**77.6**	77.9	*85.3*	Mixed
Natalizumab^a^	79.6	**83.7**	86.2	**80.9**	Chimeric
Ranibizumab^b^	69.4	**75.8**	80.0	*87.4*^e^	Mixed
Bevacizumab^b^	71.4	**76.8**	81.1	*88.4*^e^	Mixed
Eculizumab^a^	72.4	**83.7**	81.1	**84.2**^e^	Chimeric
Efalizumab^b^	68.4	**76.5**	83.2	*89.5*^e^	Mixed
Omalizumab^b^	69.7	**78.6**	77.8	*86.9*^e^	Mixed
Alemtuzumab^a^^,^^d^	62.0	**73.7**	88.4	*86.3*^f^	Mixed
Palivizumab^a^	78.8	*87.9*	68.5	**81.9**	Mixed
Daclizumab^a^	81.6	**82.7**	74.2	**84.0**	Chimeric

a Humanized by grafting CDRs onto single selected VH and VL region genes;

b Humanized by grafting CDRs onto consensus VH and VL region genes;

c Humanized by grafting CDRs onto a small library of VH and VL region genes.

d Compared to rat sequences rather than mouse.

e top “hit” macaque.

f top “hit” species of origin.

### Influence of the Foreign Sequence on Humanization Outcome

**Figure 1.**
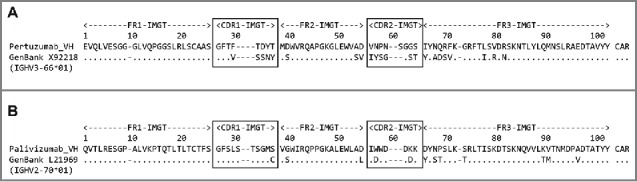
BLAST ^8^ identity analysis of (a) pertuzumab VH chain and (b) palivizumab VH chain. The humanized sequences are compared to their closest human germline counterparts (both GenBank accession numbers and IMGT® identifiers are shown). Identity, indicated by dots, and sequence differences are shown below the humanized antibody sequences. IMGT® numbering and CDR definitions (boxed) ^7^ are used with sequence gaps indicated by dashes.

The extent to which an antibody can be humanized and still fall within the definition of humanized is affected largely by the degree of identity of the CDRs of the foreign antibody to those of the closest human germline. For example, pertuzumab^31^ would now receive a chimeric designation since, although both chains show greater identity to germline human variable domain sequences than to mouse germline sequences, they are below the 85% threshold. Analysis of the heavy chain by BLAST^8^ (**Fig. 1**(a)) reveals a poor level of conservation between the mouse CDRs (IMGT® definition) and their equivalent residues in the closest human germline sequence. Given that, as a minimum, transfer of the complete CDRs plus adjacent conserved residues (i.e., residues 26, 39, 55 and 66; note that IMGT® numbering^7^ is used throughout) is required to recapitulate antigen binding, this means that a minimum of 13 residues will already differ between the humanized variant and the closest human germline, i.e. 87.6% identity. This leaves only 2 further residues that can be manipulated outside the IMGT® CDRs without falling below the 85% threshold, whereas a further 9 murine residues have been retained to maintain affinity based upon experimental evidence^31^ since simple transfer of CDRs (Kabat definition) yielded an antibody that failed to bind to antigen. Contrast this with palivizumab,^34^ which has 87.9% variable heavy chain human germline identity (**Fig. 1**(b)), where only 5 residues differ between murine and human IMGT® CDRs (plus adjacent residues) and there are 7 additional framework changes. Even if palivizumab contained 9 framework changes (as in pertuzumab), it would still fall within the WHO INN definition of a humanized antibody. In this case, it is not the framework engineering that made the difference, but the fortunate coincidence that the murine and human CDRs were more similar.

This example illustrates how the unique sequence of the initial foreign antibody can influence the humanization design process and determine how the WHO INN Expert Group interpret its categorization. This is underlined by the work of Abhinandan and Martin^41^ analyzing the “degree of humanness” of mouse sequences. This analysis revealed that there are some mouse variable gene families that can easily be humanized to similar human V-gene families even to the extent that they share sequence identity in the CDRs and would therefore likely meet the new criteria for humanized. However, there are other variable regions that are so dissimilar (our analysis indicates 8% of variable light and 6% of variable heavy mouse germline genes) that they could never meet the new criteria even if no framework residues were transplanted, nevertheless engineering of those sequences to reduce immunogenicity still makes sense for clinical development. Additional analysis of the mouse and human genomic variable gene repertoires shows that if 5 framework residues are included in the graft (or are present as natural somatic mutations), then the frequency of mouse germline sequences that can never achieve the 85% threshold increases to 64% and 35% variable light and heavy, respectively.

### Influence of framework residues on humanization outcome

As mentioned above, CDRs were originally defined by the inherent hypervariability of specific locations within the variable domain,^5^ an observation that concurred with the function of antibodies in generating a virtually limitless antigen binding repertoire. Subsequently, they were recognized as a structural feature of the antibody,^6,7^ essentially defining loops in the β-sandwich structure. Unfortunately, the structural definitions of the beginning and end of the CDRs are to some degree arbitrary, and the definition of sequence variation depends on the size of the database considered. Even a functional definition of CDRs is problematic since consideration of sequence differences between germlines (for CDR1 and CDR2), somatic mutation frequencies, or contact residues in structures of antigen-antibody complexes, will all result in slightly different definitions. Importantly, all functional definitions would change with increased knowledge of mutation data and the number of antibody/antigen complex structures determined in crystallography. The IMGT® CDR definitions^7^ are based upon strict structural classification, where the CDRs are simply regarded as being the loops connecting β-sheets. This narrow definition has been very useful in structural engineering of antibodies but fails to address the complexity of their function, and thus the impossibility of defining a clear boundary. For instance, the hypervariable residues lying outside the IMGT® CDRs but within the Kabat CDRs (i.e., light chain residues: 24–26, 39, 40, 66–69, heavy chain residues: 39, 40, 55, 66–74) may not routinely make contact with the antigen (although there are many instances where this is the case^42,43^) but they often play a critical role in determining CDR conformation, and key residues have been identified, based on antibody crystal structure data, that determine CDR “canonical structures.”^6,42^ Furthermore, additional framework residues (lying outside both CDR definitions) have also been identified that are important in maintaining CDR conformation (either Vernier Zone residues,^44^ or canonical structure residues^6,42^), and in many cases these residues overlap. Therefore, the art of antibody humanization is not just in transferring CDR residues from a donor sequence to an acceptor framework, but in identifying those additional residues that are key in supporting the correct CDR conformation for antigen engagement. Importantly, the final humanized sequence, in terms of the number of framework residues that are transferred, will depend heavily upon the sequence of the starting foreign antibody and its similarity to the closest human germline (as discussed above).

**Figure 2.**
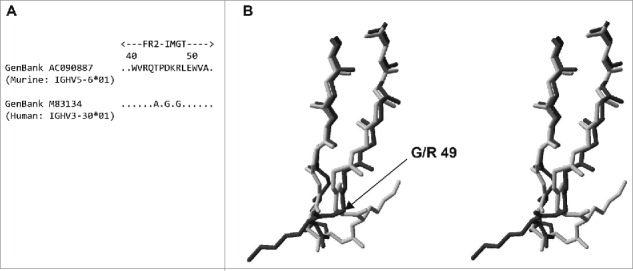
(a) BLAST ^8^ comparison of murine IGHV5-6*01 framework 2 with human IGHV3-30*01 framework 2. (b) Stereo view of an overlay of framework 2 from 3FFD (dark grey) with framework 2 of 2ADG (light grey). Only back-bone atoms are shown with the exception of lysine 48. The location of glycine/arginine 49 is indicated. The structures were analysed using Swiss-PDB Viewer ^47^ (http://www.expasy.org/spdbv/).

We and others have a large body of experimental data showing that transfer of IMGT® CDRs plus conserved flanking residues from a foreign antibody to a human template is not usually sufficient to recapitulate antigen binding and, most frequently, results in undetectable binding. Expanding the transferred residues to include the Kabat CDR residues usually restores some degree of binding (and in some cases binding equivalent to the starting antibody), but in most cases varying numbers of additional framework residues are required. Structural analysis of the foreign and human template sequences reveals the critical involvement of additional residues not included in CDRs, such as Vernier Zone or canonical structure residues, and occasionally other framework residues. For example, a murine antibody that contained a framework 2 identical to mouse germline sequence IGHV5–6*01 (GenBank accession no.: AC090887) was compared to human germline sequence databases and the human germline IGHV3–30*01 (GenBank accession no.: M83134) was used as template for humanization (**Fig. 2**(a)). Searching of the RCSB Protein Data Bank (www.rcsb.org) identified structure 3FFD^45^ as representative of the mouse framework 2, and 2ADG^46^ as representative of the corresponding human framework sequence. Comparison of the framework 2 structures (**Fig. 2**(b)) demonstrated that the identity of residue 49 dramatically affected the conformation of the C-C’ turn. Glycine 49 in 2ADG (light grey chain in**Fig. 2**(b)) was able to adopt “forbidden” dihedral angles, which resulted in the loop and associated lysine 48 (side-chain shown) being twisted through almost 180° compared to its location in 3FFD (dark grey chain) where arginine 49 adopted “permitted” dihedral angles. Although distant from the paratope, this displacement of the lysine 48 side-chain was predicted to affect the interaction of the variable heavy domain with the variable light domain and consequently antigen binding. Synthesis of humanized antibodies containing either glycine or arginine at position 49 revealed that the variant containing the human amino acid glycine 49 had a 10-fold reduction in affinity compared to the variant containing the murine amino acid arginine 49. This example serves to reinforce the point that transfer of CDRs is not normally sufficient to retain antigen binding in humanized antibodies and varying numbers of framework residues need to be taken into consideration based upon factors other than direct involvement in antigen binding.

Thus, the fine-tuning of antibody/antigen interactions during humanization is achieved by framework mutations, and this situation is fundamentally similar in technologies used for generating “fully” human antibodies. The isolation of antibodies from synthetic libraries encoding human antibodies, almost always includes mutations outside the CDRs, either originating from the library design or generated in subsequent affinity maturation steps. Furthermore, as shown in the examples of the anti-HIV antibodies, somatic hypermutation is part of the underlying process for generating potent humoral immune responses in the human immune system as well as in transgenic mice expressing human IgG repertoires; framework mutations are a key element of this.^29^ In other words, many binding problems cannot be solved by antibodies with ≥ 85% identity to the closest human variable region germline. The immune system knows this, as does the experienced antibody engineer.

## Conclusions

In summary, the current WHO INN guidance^4^ for the definition of chimeric, humanized and human antibodies is flawed for the following reasons:

- The new definitions are inconsistent with several decades of precedence in naming antibodies in the scientific literature as well in many cases as with previously assigned INN names for antibodies.
- The 85% sequence threshold is arbitrary. Moreover, there is no scientific evidence that this particular threshold equates with an improved therapeutic outcome, such as reduced immunogenicity. Indeed many sequences, whether humanized (due to the need to include foreign framework residues) or derived from humans (as a result of somatic mutation), fall below this threshold.
- The exclusion of the J-region from the INN defining process is an additional flaw as this region forms a critical part of the antibody variable domain.
- There is no specific definition of what constitutes a human antibody and what differentiates it from a humanized antibody. Any consideration of the methodology used to derive the protein would defeat the purpose of the INN reform to only regard the final sequence.
- No matter what, if any, INN suffix substem system is employed, sufficient information for antibody engineers to apply “the tools of their trade” is required. The current definitions lack sufficient information that would enable a researcher to determine how an antibody would be categorized.
- The definitions are applied retrospectively and without any notice period so antibodies designed and engineered under the previous WHO INN definition of what “humanized” means are now subject to the new definition. It is critical that a re-definition of the criteria applied should be backward consistent with the existing nomenclature as well as the scientific and clinical literature.

Technologies for antibody generation are continuously developing, and the once simple and clear distinction between chimeric and humanized or human has become increasingly blurred. Antibodies can be made from semi-synthetic and synthetic libraries, from transgenic animals, from humanization techniques as well as by all manners of other innovative methods. Thus, in principle the attempt to disregard the technology used in the generation of the antibody and base its INN on the final sequence makes sense.

While the WHO must be commended for continuously adapting the INN rules and recognizing technological progress, it appears that the latest definitions may have inadvertently lead to major intrinsic inconsistencies, and thus to unintended confusion in the field. It appears questionable whether a classification, once meant to delineate “self” from “almost self” to “non-self,” is still useful at a time when there is almost a continuum of sequences and of technologies for generating them. When this is contrasted to the almost complete ignorance of the exact level of immunogenicity that a “human-like” antibody will have before going into a clinical trial, it seems almost presumptuous to classify antibodies in a way that will potentially be considered by prescribers, who may lack technical knowledge, as simply good and bad.

A new INN system for antibodies is urgently needed to accommodate all technologies for antibody discovery as well as the myriad of new antibody-related formats. Close dialog between the WHO INN Expert Group and key stakeholders is highly desirable in developing a new INN system that effectively serves the needs of researchers as well as healthcare professionals. A single substem encompassing all engineered antibodies (perhaps -sy- for “synthetic” or -e- for engineered to replace and broaden -zu-) would greatly simplify the system, or perhaps the time has come to simply call them “-mab.” Either of these appears a better solution than an arbitrary categorization that lacks any scientific foundation.
